# Low adherence to topical therapy in adults with atopic dermatitis: A cross-sectional study

**DOI:** 10.1016/j.jdin.2026.02.009

**Published:** 2026-03-06

**Authors:** Benjamin Kendziora, Irma Kupf, Luis Messner, Pia-Charlotte Stadler, Michael J. Flaig, Laurie Eicher, Andreas Wollenberg

**Affiliations:** a Department of Dermatology and Allergy, University Hospital, LMU, Munich, Germany; b Department of Dermatology, Thalkirchner Street Hospital, Munich Municipial Hospital Group, Munich, Germany; c Department of Dermatology and Allergy, University Hospital, University of Augsburg, Augsburg, Germany

**Keywords:** atopic dermatitis, cross-sectional studies, emollients, epidemiology, medication adherence, patient preference, personality, steroids, topical administration, treatment adherence and compliance

To the Editor:

Poor adherence to topical therapy remains a challenge in atopic dermatitis and dermatology more broadly. While treatment burden is well described for systemic therapies (“pill burden”), less is known about the burden of topical treatments. We examined adherence to topical therapy, associated patient-related and treatment-related factors, and patient-reported preferences and perceived barriers and facilitators.

In this exploratory cross-sectional study performed in a tertiary outpatient clinic, physicians assessed adherence to emollients, active topicals, and systemic therapy using a 0 to 100 visual analog scale based on patient self-report, reflecting the estimated proportion of prescribed applications performed. Associations between adherence and sociodemographic, psychological, disease-related, and treatment-related factors (Supplementary Tables I-IV, available via Mendeley at https://data.mendeley.com/datasets/59zh6fj7ww/1) were examined using multivariable regressions, and chi-squared tests with Bonferroni correction were used to analyze patient preferences.

Mean adherence was lowest for active topical therapy (54%) compared with emollients (65%; *P* = .018) and systemic therapy (95%; *P* < .001). In multivariable analyses, lower adherence to emollients was independently associated with lower conscientiousness, higher extraversion, and concomitant systemic or active topical therapy (Table I). Lower adherence to active topical therapy was associated with as-needed rather than daily prescription, concomitant systemic therapy, male sex, and younger age (Table II). Patients most frequently preferred creams (67%) over lotions (18%; *P* = .002) and ointments (16%; *P* < .001) and finished (83%) over compounded preparations (17%; *P* < .001). Slow absorption (62%) and time demands (51%) were the most reported barriers, whereas simplified regimens (36%) and digital tools (25%) were the most reported facilitators (Supplementary Tables VII and VIII; Supplementary Figs 1 and 2, available via Mendeley at https://data.mendeley.com/datasets/59zh6fj7ww/1).

**Table I:** Regression estimates of the multivariable regression on associations to the adherence to emollient-based topical therapy

Predictor∗	Estimate	SE	Statistic	*P* value
Intercept	71.725	22.372	3.206	.002
BFI-10, conscientiousness	11.935	3.336	3.578	.001
BFI-10, extraversion	−7.719	2.562	−3.013	.004
Prescribed systemic therapy: yes	−16.185	5.701	−2.839	.006
Prescribed active ingredient–containing topical therapy: yes	−26.448	11.563	−2.287	.026
Gender: female	9.439	5.755	1.64	.106
Age, y	0.085	0.191	0.448	.656

*BFI-10*, Big Five Inventory; *SE*, standard error.

∗ Univariable regressions including sociodemographic, psychological, disease-related, and treatment-related factors were performed first (Supplementary Table V, available via Mendeley at https://data.mendeley.com/datasets/59zh6fj7ww/1), and variables with *P* < .20 were entered into the multivariable model. Age and sex were included and retained in the multivariable model irrespective of statistical significance, whereas other variables were sequentially removed if *P* > .05.

**Table II:** Regression estimates of the multivariable regression on associations to the adherence to active ingredient–containing topical therapy

Predictor∗	Estimate	SE	Statistic	*P* value
Intercept	50.401	8.149	6.185	0
Prescribed systemic therapy: yes	−11.896	5.457	−2.18	.033
Schedule of application of prescribed active ingredient–containing topical therapy: as-needed (daily as default)	−44.196	8.49	−5.205	0
Schedule of application of prescribed active ingredient–containing topical therapy: both daily and as-needed (daily as default)	−14.867	5.501	−2.702	.009
Gender: female	11.441	5.436	2.105	.039
Age, y	0.395	0.18	2.196	.031

*SE*, Standard error.

∗ Univariable regressions including sociodemographic, psychological, disease-related, and treatment-related factors were performed first (Supplementary Tables VI, available via Mendeley at https://data.mendeley.com/datasets/59zh6fj7ww/1), and variables with *P* < .20 were entered into the multivariable model. Age and sex were included and retained in the multivariable model irrespective of statistical significance, whereas other variables were sequentially removed if *P* > .05.

Lower adherence among patients prescribed active topical therapy on an as-needed basis compared with daily schedules may reflect greater difficulty in integrating less structured regimens into daily routines, but could also be confounded by other factors, such as physicians’ expectations regarding adherence or disease severity.

The association of higher conscientiousness and introversion, 2 of the “Big Five” personality dimensions, with better adherence to emollients is consistent with broader health behavior research on adherence to medical recommendations.^1^^,^^2^ Associations of female sex and older age with higher adherence have also been reported, although findings are inconsistent.^3^^,^^4^ These patterns highlight a need for treatment regimens that accommodate demographics and behavioral profiles.

When multiple topical formulations are clinically appropriate and no compelling medical indication favors a specific option, patient-reported preferences may be considered. In this study, creams were more frequently preferred than lotions or ointments and finished over compounded preparations.

Key limitations include the small sample size, reliance on physician-rated adherence based on patient self-report, which may overestimate adherence,^5^ incomplete exposure to all topical formulations in the assessment of patient preferences, and the lack of measurement of patient-physician relational factors that may influence adherence. The cross-sectional design precludes causal inference.

In conclusion, adherence to topical therapy in atopic dermatitis is substantially lower than adherence to systemic treatment. Lower adherence to topical therapy is associated with as-needed prescription, concomitant systemic therapy, lower conscientiousness, higher extraversion, younger age, and male sex. Patients more frequently prefer creams over lotions or ointments and finished over compounded preparations.

## Conflicts of interest

None disclosed.
